# Real Time Tracking of Magmatic Intrusions by means of Ground Deformation Modeling during Volcanic Crises

**DOI:** 10.1038/srep10970

**Published:** 2015-06-09

**Authors:** Flavio Cannavò, Antonio G. Camacho, Pablo J. González, Mario Mattia, Giuseppe Puglisi, José Fernández

**Affiliations:** 1 Osservatorio Etneo, Istituto Nazionale di Geofisica e Vulcanologia, Catania, Italy; 2 Institute of Geosciences (CSIC-UCM), Madrid, Spain; 3 COMET. Institute of Geophysics and Tectonics, School of Earth and Environment, University of Leeds, Leeds, United Kingdom.

## Abstract

Volcano observatories provide near real-time information and, ultimately, forecasts about volcano activity. For this reason, multiple physical and chemical parameters are continuously monitored. Here, we present a new method to efficiently estimate the location and evolution of magmatic sources based on a stream of real-time surface deformation data, such as High-Rate GPS, and a free-geometry magmatic source model. The tool allows tracking inflation and deflation sources in time, providing estimates of where a volcano might erupt, which is important in understanding an on-going crisis. We show a successful simulated application to the pre-eruptive period of May 2008, at Mount Etna (Italy). The proposed methodology is able to track the fast dynamics of the magma migration by inverting the real-time data within seconds. This general method is suitable for integration in any volcano observatory. The method provides first order unsupervised and realistic estimates of the locations of magmatic sources and of potential eruption sites, information that is especially important for civil protection purposes.

Multivariate collected data and robust mathematical models are today becoming crucial for providing effective volcano monitoring and early warnings to civil authorities. Volcano warning systems are typically specific for each volcano and are usually based on thresholds of some well-defined indexes based on the recorded signals^1^,^2^,^3^,^4^,^5^. Indeed, in the last decade, many techniques have been developed to monitor the state of active volcanoes in real time^6^. Among all the different kinds of measurable parameters, ground deformation is one of the most reliable components for any volcano monitoring strategy, since surface deformation directly reflects volcanic processes at depth (i.e. pressure and/or mass changes) transmitted to the surface through the mechanical properties of the crust^1^,^2^,^7^. Since mass changes produce variations in the natural gravity field, gravity changes are often introduced in ground deformation monitoring systems^8^,^9^. Hence, surface deformation and gravity changes in volcanic areas are important indicators of changes in the equilibrium conditions of structures and magma sources, and their correct interpretation may be a valid aid in hazard evaluation.

To this end, modeling of deformation caused by volcanic sources has achieved significant progress in the last decades. A number of analytical and numerical mathematical models, with many source geometries, are available in literature, and can be used to fit ground deformation and gravity data to infer source location, depth and density^8^,^9^,^10^,^11^,^12^. Usually, analytical magma source models represent mathematical abstractions based on strong assumptions (e.g. a-priori definition of the source shape, use of geometrically simple geometries, and selection of medium characteristics) that make the set of differential equations describing the problem tractable. Sometimes the analytical approach must be limited to a very simplified and rough approximation of the real source. Nevertheless, analytical models may be useful in certain applications where priority is given to the fast computation of inverse solutions.

On the other hand, numerical Finite Element (FE) modeling allows realistic features such as topography and crustal heterogeneities to be included. However, FE modeling requires large computation resources and it is still very time-expensive to solve the inverse problem with FE models for real-time monitoring purposes, i.e. it can take several hours of computation to invert data in order to estimate the source parameters^13^,^14^.

In an operative real-time monitoring system, minimizing the delay in the estimation of location and characteristics of the shallow magma source and their evolution in time is the desired goal. Minimum delay in inferring the possible location and timing of an impending eruption would provide critical time for decision makers. Indeed, the fast response of a magma source model can be crucial in early warning for mitigating the possible dramatic consequences of an eruption near populated zones. In this regard, the use of an analytical model for data inversion could speed up the source estimation. Nevertheless, it is still necessary to impose assumptions on the a-priori source geometry, though unfortunately, such selection is often highly unconstrained due to the complexity and lack of knowledge of volcano plumbing systems.

Our goal is to estimate in real time, for a given volcano showing signs of unrest, the location, size and volume distribution of the deformation source and to track those parameters through time without any a-priori assumption on the source geometry. Our method seeks to delimit the area with the highest likelihood of being the location of an opening fissure or vent for the impending eruption. Other authors have already proposed real-time techniques for empirical early warning systems. *Endo and Murray*^15^ used raw real-time seismic amplitude measurement to predict the volcanic eruptions that occurred at Mount St. Helens between 1985 and 1986. *Aiuppa et al*.^16^ considered the real-time observations of H_2_O, CO_2_, and SO_2_, combined with well-constrained models of degassing, as good features for Mount Etna eruption forecasting. This method unfortunately provides only the timing of eruption. *Aki and Ferrazzini*^17^ used the seismic monitoring and modeling of Piton de la Fournaise volcano to predict the magma transport process in real time. *Ji et al*.^6^ applied the Targeted Projection Operator (TPO)^18^ to continuous GPS data from Long Valley Caldera and concluded that this methodology could be used for near real-time monitoring with 1-day latency for rapid processing and 14–20 days latency when final IGS GPS orbits were used. They used an analytical Mogi^19^ source as a reference^20^ to interpret the observed velocity field. *Crescentini et al*.^21^ proposed a numerical package to invert geodetic data for different kinds of analytical sources embedded in an elastic-gravitational layered half-space^8^. Their developed code is fast enough to be suitable for near real-time applications in alert systems, running an inversion every few minutes to some hours. *Vasco et al*.^22^ proposed a methodology for three-dimensional reconstruction of magma reservoir geometry from InSAR data and opened a new research branch called geodetic imaging. The methodology has recently been applied to Campi Flegrei caldera (southern Italy)^23^. It is worth noting here that for all these early warning methodologies, the time needed to invert data and infer the magmatic source parameters, must always take into account the time delay necessary to obtain/compute coordinate changes (or any other parameter processing).

In this paper, we apply a new approach to model volcanic sources and track their evolution in time. The proposed approach combines the advantages of fast calculation of the analytical models and adds the capability to model free-shape distributed sources. The objective is to address the limitations in the inversion process dictated by the running time and the a priori assumption on the shape of the intrusion or magma chamber in order to produce a useful real-time that only adds seconds to the time needed to obtain displacement data. We use the inversion methodology proposed by *Camacho et al*.^24^. This inversion methodology performs a non-linear inversion of the displacement data, producing extended bodies^25^ with a free geometry (see *Methods*) within seconds (less than a minute) in a fully automatic way, i.e. without requiring any user input. To demonstrate the applicability and results obtained using this methodology in real-time tracking of magmatic intrusions using ground deformation data we have selected the main eruption at Mount Etna, Sicily, Italy, in 2008 as a test case. The 13 May 2008 flank eruption on Mt. Etna occurred on the upper eastern flank after an intense recharge phase beginning in early 2007, interrupted by seven fire fountain episodes at the New South East Crater (NSEC), the last of which took place on 10 May 2008. The intrusion produced large ground displacements captured by the volcano CGPS network^26^. Because of the magnitude of the event and the quality, quantity and variety of available data, this eruption is a good benchmark to test new volcano modeling techniques. In particular, we model the ground deformation pattern associated to the co-intrusion dyke propagation in the shallower part of the northern sector of Mt. Etna. We use the high rate GPS (HRGPS) data coming from 10 stations (Fig. 1a) from the Etnean GPS permanent network (Etn@net) to examine the dynamics of the magma intrusion^27^. We simulated a real-time scenario by computing HRGPS solutions from the raw data and running the inversion code, thus simulating a real processing and inversion stream during the eruption process. We started our real-time analysis the day before the eruption when GPS signals showed some transients, though it should be noted that the shallow co-intrusion period lasted from the 8.30 am UTC^26^ (when the seismic swarm started) on 13 May 2008 to 04.00 pm UTC the same day (when almost all the seismic activity had ended). The real effusion^26^ phase, together with lava fountaining, started at 9.30 am UTC, as revealed by Meteosat Second Generation satellite. The fountaining stopped at around 11.00 am UTC^28^. This implies that the early warning window to localize the impending eruption site was only 1 hour (from 8.30 am to 9.30 am UTC).

## Results

**Figure 1:**
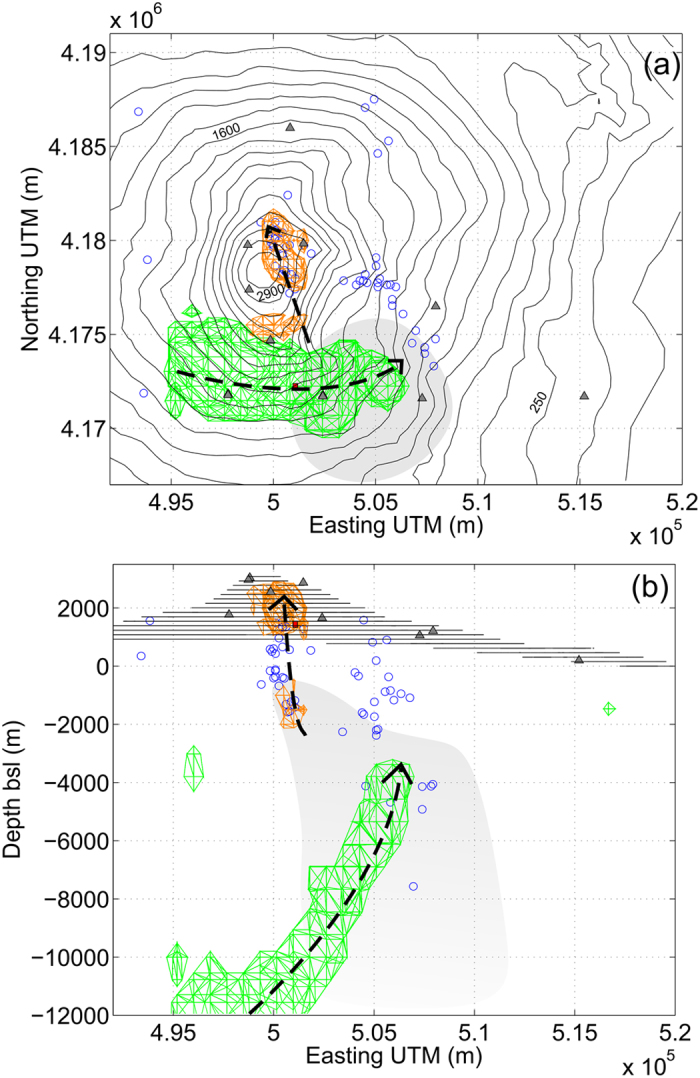
**Planar view (a) and EW vertical cut view (b)** of the main elements from the real-time modelling process covering the inflation period and the eruption on 13 May 2008, and possible connections between the source bodies and the location of the earthquakes (blue circles) for 12–14 May 2008. Contours correspond to surface topography. Gray triangles indicate the location of the GPS stations. Shaded gray area outlines the position of the High Velocity Body (HVB)^29^. In green, the cumulated sources during the year-long recharging phase and in orange the sources active during the day of the eruption, some of which are shown in the snapshots of Fig. 3. We suggest fast magma ascent from the reservoir level (2–3 km depth bsl) along paths indicated by the sources geometry and the earthquake locations (dashed lines in the figures). The figure was made using MATLAB^©^.

**Figure 2:**
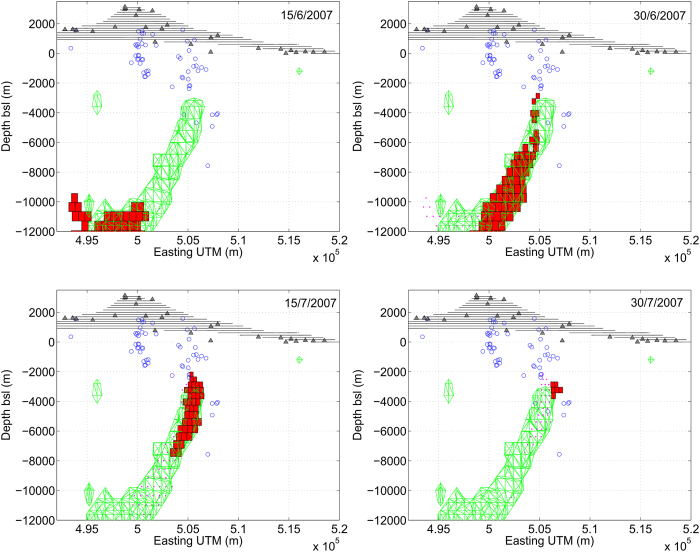
Model for the inflation phase. Cumulated magmatic body (in green) modelled for the period 1 January 2007 to 12 May 2008 and time evolution (in red) for the sub-period that corresponds to the months of June and July 2007, when the system was subject to a recharging episode, which appears as an ascending high pressure body. Parallelepiped sources represent active point pressure sources within the same volume. Blue circles correspond to location of the earthquakes for 12–14 May 2008. The figure was made using MATLAB^©^.

Before testing the real-time response of the inversion procedure, we used the proposed methodology to analyze the inflation phase preceding the 13 May 2008 eruption. We modeled the time evolution of the magmatic source for the period 1 January 2007 to 12 May 2008 by considering 15 day-intervals (see Figs. 1 and 2). We found that during the months of June and July 2007, the system was subject to recharging episodes characterized by an ascending high pressure body. As shown in Fig. 2, the modeled body ascended from a depth of about 10 km, to reach a level of about 2–3 km depth (b.s.l.). The modeling allows estimating the rate of magma ascent and suggests an approximate 3D geometry. We assume that an a-priori estimated knowledge and interpretation of mid-term (weeks-to-months) ground deformation signals are routinely obtained in most volcano observatories. Such estimated models account for typical monitoring resources, which include a weekly report of volcanic activity, integrating the available geodetic data on those time scales.

**Figure 3:**
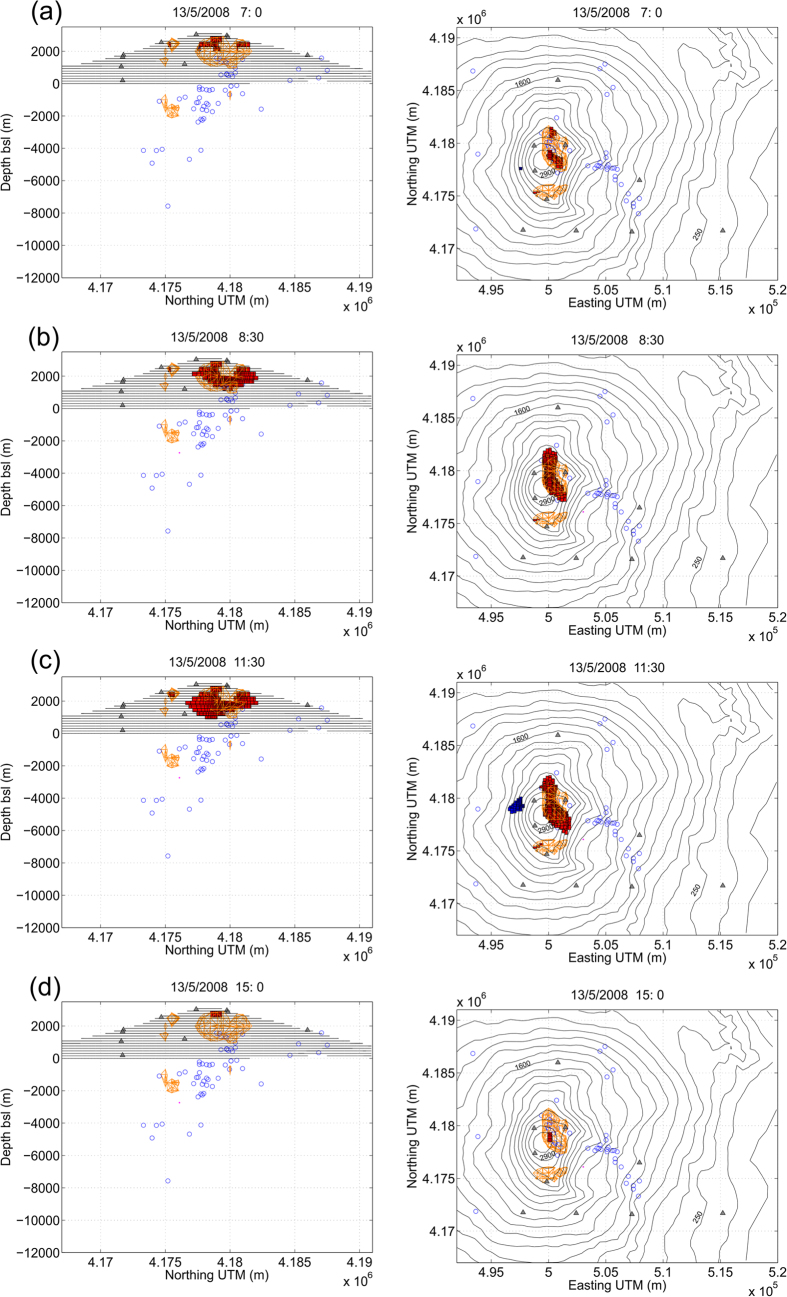
Time sequence of pressurized source models (described by aggregation of parallelepiped cells which map active point pressure sources) corresponding to key instants from the real-time inversions during the 13 May 2008, eruption of Mount Etna volcano at different UTC times: (**a**) at 7:00, (**b**) at 8:30, (**c**) at 11:30, and (**d**) at 15:00. Red volumes are positive-pressure sources while blue volumes are negative-pressure sources. Structures marked in orange correspond to the cumulated sources describing the main source structures across the sequence. Contours correspond to surface topography. Gray triangles indicate the location of the GPS stations. Blue circles correspond to location of the earthquakes for 12–14 May 2008. The figure was made using MATLAB^©^.

Nine months after the deep magma ascent, a dyke intruded in the North-East sector of the Etna summit area. The simulated real-time inversion produced a time series of 3D body sources shown in Fig. 3. In particular, in Supplementary Videos we observe that in the evening (20–22 UTC, all the times are in UTC hereinafter) of the day before the eruption, a vertically elongated source from 2 km bsl to 2 km asl identifies a magma batch likely migrating towards the surface. Starting from 6.00 am of the day of eruption, a large positive pressure source located at about 2.5 km asl in the NE sector of the summit area triggered the dyke intrusion at 7.00 am towards the shallow complex source in the NW-SE direction (see Fig. 3, panel **a**). The latter source reached its maximum expansion in that direction at 11.30 am (Fig. 3, panel **c**) and contracted only after 12:30 pm. This activity follows exactly the sequence of events and models reported in previous studies^26^,^28^. The estimated pressure sources show, minute by minute, that most of the recorded deformation corresponds to a shallow dilation process located close to the NE sector of the summit area, just where the eruption fracture opened.

**Figure 4:**
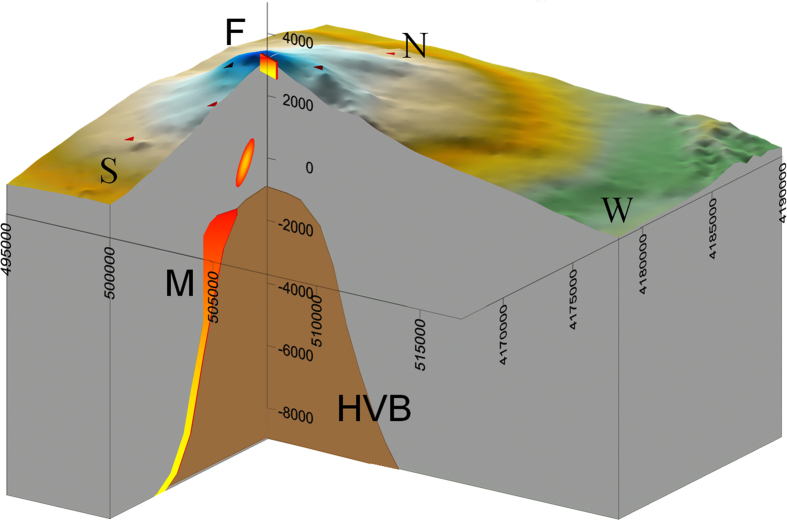
Graphic summary of the sources modeled in the studied 2008 Mt. Etna eruption. HVB: High-velocity body, F: the dyke as modelled by the present study and previous works^23^, M: main source body for the inflation phase preceding the eruption. UTM coordinates and depth are expressed in meters. The azimuth view is 40°.

Figure 1 shows the traces of the major sources that emerged from the inversion for both the studied time scales (long and short) under consideration together with seismicity during these periods. The uncertainty of the source location was estimated at about 500 m in the horizontal component and 900 m in the vertical component. Thus, by considering the associated errors of the earthquakes (200–300 meters in all the 3D components) recorded by the local permanent seismic network managed by INGV (http://www.ct.ingv.it), from Fig. 1 we can estimate the earthquake swarm to be co-located with the estimated magma sources during the intrusion phase. It is worth noting that all the identified elongated model sources seem to occur near the periphery of the High Vp Body (HVB)^29^,^30^. A comparison between the shape and the position of the volumes estimated here and the ones modelled by *Bonaccorso et al*.^31^ for the period 1993–2000 suggests that there is a magma reservoir at about 2 km bsl and a vertically elongated source located in the south-western border of the HVB, where magma migrates towards the surface. Figure 4 provides a 3D-view sketch summarizing the results.

The goodness-of-fit for the estimated time varying model was assessed by computing the root mean square error (RMSE) between the measured and the modelled displacements. See Supplementary Fig. S3 for a graphic comparison between the observed and modelled EW displacement velocities time series during the day of eruption for all selected GPS stations. Discrepancies between observed and modelled values are in the order of 0.2 and 0.4 mm/day for horizontal and vertical components displacements, respectively.

Some spurious sources appear scattered around in the domain (see e.g., Fig. 3 and Supplementary Videos). They are usually due to the residuals in the data (non-ideal network topology configuration, noise measurements, and errors in the parameter settings) that the algorithm attempts to account for. A suitable trade-off between good data fit (with possible aggregation of spurious sources) and good model stability (with poor data fit and too simple model geometry) is obtained by analyzing the autocorrelation of the resulting data residuals (see *Camacho et al*.^24^ for additional information). Nevertheless, spurious sources are usually easily distinguishable, but sometimes they aggregate, forming a volume that may appear as a real source. In such cases, considering the spatial location, the size, the shape and their temporal evolution, they can usually be identified and discarded as real sources of deformation.

**Figure 5:**
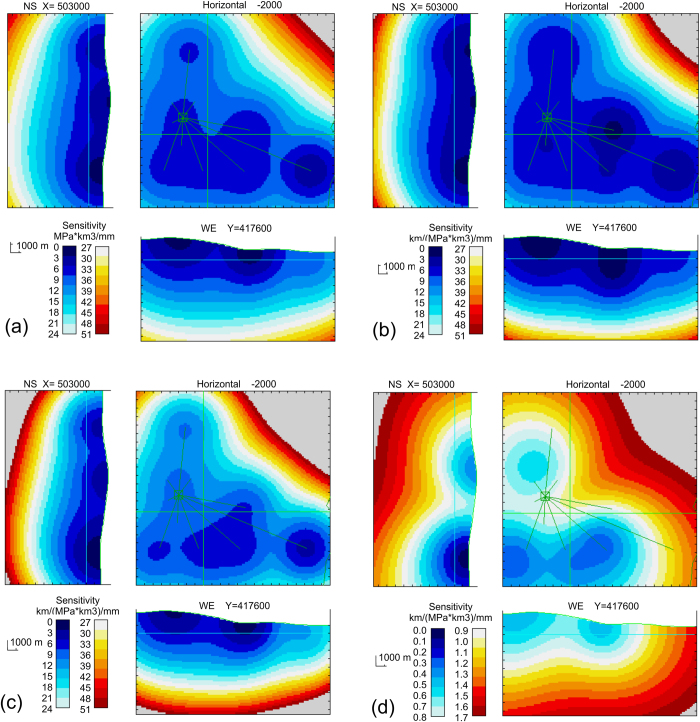
3D uncertainty maps for the considered GPS network of 10 stations. It represents respectively the pressure changes (**a**), the depth changes (**b**), and the horizontal deviations (**c**) able to produce a surface deformation with quadratic mean value 1 mm. Map (**d**) represents the minimum horizontal deviation for an isolated body of 1 km^3^ with a pressure change given in panel (**a**) to produce a deformation at the GPS stations with r.m.s. magnitude of 1 mm.

In order to quantify the reliability of the solutions obtained with our method, we have carried out an uncertainty analysis. In Fig. 5a we show how the sensitivity to pressure changes decreases with depth and with the distance to the stations. In fact, the required pressure (for a 1 km^3^ body) to produce a particular magnitude of surface deformation at the current network stations (root mean square 1 mm) increases with depth and distance to the stations. For deep and distant pressure elements in the inverse model, the relative uncertainty increases with a pattern similar to that of Fig. 5a.

Figures 5b,c show the 3D uncertainty distribution for vertical and horizontal displacements of the isolated pressure sources. It also is valid for some geometrical components or details of extended sources. These maps show the location values (in km), for a source volume 1 km^3^ and a pressure value 1 MPa, required to produce a particular surface deformation level (1 mm, r.m.s. value). They can be interpreted as showing the pattern of relative uncertainty for the geometrical aspects (depth and horizontal location) for the inverse model. Also in this case, the displacement uncertainty pattern is heavily dependent on the pattern of the uncertainty for the source intensity (pressure and volume) of the assumed source element. A more valuable uncertainty map for model geometrical aspects is obtained by assuming equally sensitive source elements (according to the pattern of Fig. 5a). Fig. 5d shows the sensitive 3D pattern for displacements for a volume of 1 km^3^, but assuming pressure values increasing with distance according to Fig. 5a. These last model uncertainties show a different pattern, essentially due to geometrical aspects. Further details on uncertainty analysis can be found in the Supplementary material. The uncertainty analysis shows the power resolution of the GPS network for the proposed source estimations. The uncertainty maps show that the sources are well-resolved in the volumes where they have been estimated in Figs. 1, 2, 3, thus making the results more reliable.

## Discussion

The main feature of the proposed approach is its ability to instantaneously provide a distribution of pressure sources for real-time volcano monitoring purposes. The approach overcomes the limitations of fixed-shape analytical models and the time-consuming nature of numerical approaches based on finite elements, providing a tool to infer the timing and location of the potential impending eruption, thus helping to activate warning procedures in good time. It may therefore be a crucial tool for civil protection purposes. For example, during the first hours of the studied 2008 Etna eruption, bad weather conditions prevented researchers from identifying the location of the ongoing eruption. However, the use of a tool like the one proposed here could have assisted researchers during the peak of the crisis by helping define the magma location in real time and infer the area of the opening eruptive fracture.

The hypothesis of homogeneous elastic medium (see *Methods*) does not appear to affect in any critical way the quality of the test results considered here, as was expected with the time periods used^8^,^32^. They show good agreement with previous results described in literature, e.g. the shallow NW-SE oriented source described above overlaps the previously modeled dyke^26^.

Tracking the evolution of the magmatic source beneath the volcano surface in real time has always been a challenge for researchers working in modern volcanic surveillance. The proposed methodology is able to estimate, in real time, the location, size and shape of the active magma source in volcanic areas and can track those parameters through time, without any a-priori assumption on the source geometry. This information can be the basis for forecasting activity in a monitoring and hazard assessment system to assist decision makers during a volcanic crisis. To this end, the presented test case showed the effectiveness of the proposed methodology for one of the best monitored active volcanoes on Earth. Mt. Etna represents a significant hazard to the local population living in neighboring areas and to buildings and infrastructure. The methodology, though successfully applied here to a Mt. Etna eruption, can be tuned and used in all active volcanic areas. Compared to other approaches in volcano monitoring by means of geodetic data, the advantages of the proposed tool are: (1) the speed of data inversion (few seconds); (2) the absence of assumptions on the shape of the source, (3) obtaining a 3D geometry of the magmatic sources and their time evolution; and (4) the easy implementation of the tool (it runs on a common personal computer without any special requirements).

## Methods

In our test cases we reproduced the real-time scenario of the eruptions. Though the test data were processed after data collection (post-processing), real-time operation was simulated. Epochs of data were processed sequentially and intermediate results (epoch-by-epoch) were used for the analysis. The GPS data were collected from the network dual-frequency receivers and processed online with Geodetics® RTD software package with Epoch-by-epoch algorithm^33^,^34^ to obtain a solution of position every second for all the measured points. The solutions then were sent to a post-processing module to improve their reliability. In particular, in this module, the outliers were discarded by using an inter-quartile range (IQR) filter and filtered for the multipath noise (due to multi-path ground reflections of GPS signal) following the technique by *Nikolaidis et al*.^35^. The remaining solutions were used to estimate the displacement variation in a time window of 30 minutes. Displacements were calculated by a robust linear regression^36^ of the GPS solutions in the 30-minutes window. We chose an interval of 30 minutes as a tradeoff between the minimum response time of the warning system and the minimum level of noise in the estimated displacements (see Supplementary Figs. S1 and S2). For example, if we assume the magma started its migration toward surface at the depth of 4–6 km below the surface ascending at a rate between^37^ 0.001–0.5 m/sec, then the time required to reach the surface is from 64 days to 2.2 hours (from about 2 months to less than a day). Therefore a 30 minute-window should be useful for our purposes. Inversions were performed in a continuous way from the simulated real-time displacement data computed every 15 minutes.

The inversion methodology, described by *Camacho et al*.^24^, carries out a simultaneous non-linear inversion of both displacements and gravity changes determined by using terrestrial and space techniques, as produced by extended bodies with a free geometry in a homogeneous elastic medium. Assuming homogenous elastic conditions, the approach can determine general geometrical configurations of pressured and/or density source and/or sliding structures corresponding to prescribed values of anomalous density and pressure. These source bodies are described as aggregation of elemental point sources for pressure, density and slip, and they fit all of the available data, keeping some 3D regularity conditions about the total anomalous magnitude. The approach works in a growth step-by-step process that allows, in a fast process, to adjust very general geometrical configurations. Anomalous pressure/density changes are nearly homogeneous within the causative bodies according to some prescribed values for both magnitudes.

This inversion process has been tested by means of simulated cases^24^, e.g. considering ellipsoidal and parallelepiped bodies for anomalous pressure and density changes. Results show a good estimation of location and morphology of the simulated causative bodies. Due to the regularization condition, the morphology of the bodies is generalized, i.e., they can appear more rounded or simplified and the actual case.

There is some general uncertainty for any inversion approach with respect to the pressure contrast ∆P of the causative structure. This uncertainty is connected to the anomalous volume. To keep a similar observed effect, for small ∆P large anomalous volumes are required, and for a high ∆P the inversion process determines a smaller causative volume. By trying tentative ∆P values, we can obtain the corresponding causative structures, and select visually the optimal value. However, the location and general morphology of the causative bodies will be nearly insensitive within a range of ∆P. In the inversion of our test case we used 1 MPa as the value of pressure change, considering typical values obtained studying different eruptions for Mt. Etna volcano and from other studies of the considered test case eruption^26^. Several tests have been performed, considering different values of pressure change (0.5 to 3 MPa), that produced very similar results.

## Additional Information

**How to cite this article**: Cannavò, F. *et al*. Real Time Tracking of Magmatic Intrusions by means of Ground Deformation Modeling during Volcanic Crises. *Sci. Rep*. **5**, 10970; doi: 10.1038/srep10970 (2015).

## Supplementary Material

Supplementary Information

Supplementary Information

Supplementary Information

Supplementary Information
