# Supplementary material for: Real Time Tracking of Magmatic Intrusions by means of Ground Deformation Modeling during Volcanic Crises
Source: Sci Rep. 2015 Jun 9;5:10970. doi: 10.1038/srep10970 (PMC4460733; doi:10.1038/srep10970)
Supplement: Supplementary Information [file srep10970-s1.pdf]

Supplementary Information

Real time tracking of magmatic intrusions by means of ground deformation modeling during volcanic crises

Flavio Cannavò[1], Antonio G. Camacho[2], Pablo J González[3], Mario Mattia[1], Giuseppe Puglisi[1], José Fernández[2]

[1]Osservatorio Etneo, Istituto Nazionale di Geofisica e Vulcanologia, Catania, Italy.

[2]Instituto de Geociencias (CSIC-UCM), Madrid, Spain.

[3]COMET. Institute of Geophysics and Tectonics, School of Earth and Environment, University of Leeds, Leeds, United Kingdom.

(+) Correspondence and requests for materials should be addressed to J.F. (email: jft@mat.ucm.es)

Time scale choice

In order to choose the best window we considered all the high-rate GPS time series for the first two weeks of April 2008 when there is no evidence of real significant displacement, thus the time series are mainly pure noise.

We analysed the noise of the windowed time series of the GPS solutions (the average for the three components) by showing in Fig. S1 the standard deviation of the displacement time series calculated with windowed robust least square. We note that the increase in precision almost stops the fast decay with windows larger than 30 minutes.

Moreover, we computed the autocorrelation function for all the time series. The average function in Fig. S2 shows that after 1000 seconds (i.e. ~15 minutes) the GPS solutions are not correlated (autocorrelation always below 0.35) in time. Thus, the choice of a 30 minute-window (i.e. 1800 seconds) enables avoiding time correlated data for linear displacement estimation.

Modelled time series

We compared the observed and modelled EW displacement velocities (cm/day) during the day of eruption for all the used GPS stations (see Fig. S2). Discrepancies between observed and modelled values are in the order of 0.2 and 0.4 mm/day for horizontal and vertical components displacements respectively.

Uncertainty Analysis

To analyse the uncertainty of the results to the GPS network configuration, we carried out the same sensitivity tests reported in the manuscript by using the latest GPS network on Mt. Etna. Figures S3 to S7 correspond to results obtained with the actual GPS network made up of the current 40 stations. They show a rather different pattern, mostly corresponding to the geometrical distribution of stations. For some common areas the 40 stations sensitivity is obviously higher (smaller pressure change or smaller displacements) than that corresponding to 10 stations. This means that the more stations involved in the inflation/deflation process (i.e. measure displacements), the more resolute the model.

FIGURE AND VIDEO CAPTIONS.

Figure S1. Best windowing. In blue the mean (for all the stations and components) standard deviations, for different window sizes, of the estimated displacement time series from GPS solutions.

Figure S2. Mean autocorrelation function with shaded error bar for all the 1-Hz GPS time series. It is possible to see the steep decay of autocorrelation after lags of a few hundreds of seconds.

Figure S3. Comparison between the observed and modelled 3D displacement velocities (mm/day) during the day of eruption for all the considered GPS stations.

Figure S4. 3D uncertainty map of the GPS 40-stations network to pressure changes, as given as the pressure (MPa), within a volume of 1 km^3, able to produce a surface deformation with quadratic mean value 1 mm. It represents the minimum pressure change for an isolated body of 1 km^3 or for a section (of volume 1 km^3) of an extended body, able to produce a deformation at the GPS stations of the network with r.m.s. magnitude of 1 mm.

Figure S5. 3D uncertainty map of the GPS 40-stations network to depth changes for the pressure sources, as given as the depth change (in km), for a volume of 1 km^3 and a pressure change 1 MPa, able to produce a surface deformation with quadratic mean value 1 mm. It represents the minimum depth change for an isolated body of 1 km^3 with 1 MPa or for a section (of volume 1 km^3 and a pressure change 1 MPa) of an extended body, to produce a deformation at the GPS stations of the network with r.m.s. magnitude of 1 mm.

Figure S6. 3D uncertainty map of the GPS 40-stations network to horizontal deviation for the pressure sources, as given as the depth change (in km), for a volume of 1 km^3 and a pressure change 1 MPa, to produce a surface deformation with quadratic mean value 1 mm. It represents the minimum horizontal deviation for an isolated body of 1 km^3 with 1 MPa or for a

section (of volume 1 km^3 and a pressure change 1 MPa) of an extended body, to produce a deformation at the GPS stations with r.m.s. magnitude of 1 mm.

Figure S7. 3D uncertainty map of the GPS - 40 stations - network to depth changes for the pressure sources, as given as the depth change (in km), for a volume of 1 km^3 and a pressure change given in figure 1, to produce a surface deformation with quadratic mean value 1 mm. It represents the minimum depth change for an isolated body of 1 km^3 with a pressure given in Fig. S4 or for a section (of volume 1 km^3 and a pressure change given in Fig. S4) of an extended body, to produce a deformation at the GPS stations of the network with r.m.s. magnitude of 1 mm.

Figure S8. 3D uncertainty map of the GPS- 40 stations - network to horizontal displacements for the pressure sources, as given as the depth change (in km), for a volume of 1 km^3 and a press change given in figure 1, to produce a surface deformation with quadratic mean value 1 mm. It represents the minimum horizontal displacement for an isolated body of 1 km^3 with a pressure given in figure 1 or for a section (of volume 1 km^3 and a press change given in figure 1) of an extended body, to produce a deformation at the GPS stations of the network with r.m.s. magnitude of 1 mm.

Videos show a time sequence of the modelling results corresponding to the successive inputs of GPS data. The causative pressurized sources are described by aggregation of parallelepiped cells. Red indicates pressure increase and blue pressure decrease. Blue circles indicate the location of the GPS stations. Structures marked in yellow correspond to the more employed cells, describing a main source structure across the sequence. Contour lines correspond to outer topography. Videos have been made using MATLAB©.

There are three videos with the following contents:

V1. mov

Video showing plain views of the successive models obtained for the real-time inversion during the eruption.

V2. mov

Video showing NS vertical views of the successive models obtained for the real-time inversion during the eruption.

V3.mov

Video showing EW vertical views of the successive models obtained for the real-time inversion during the eruption.

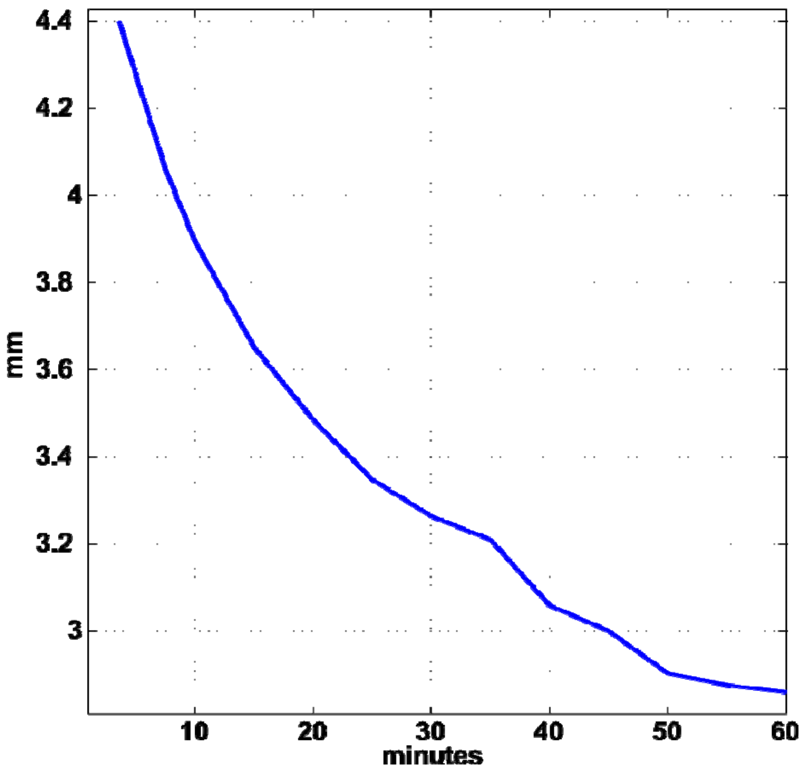

Figure S1

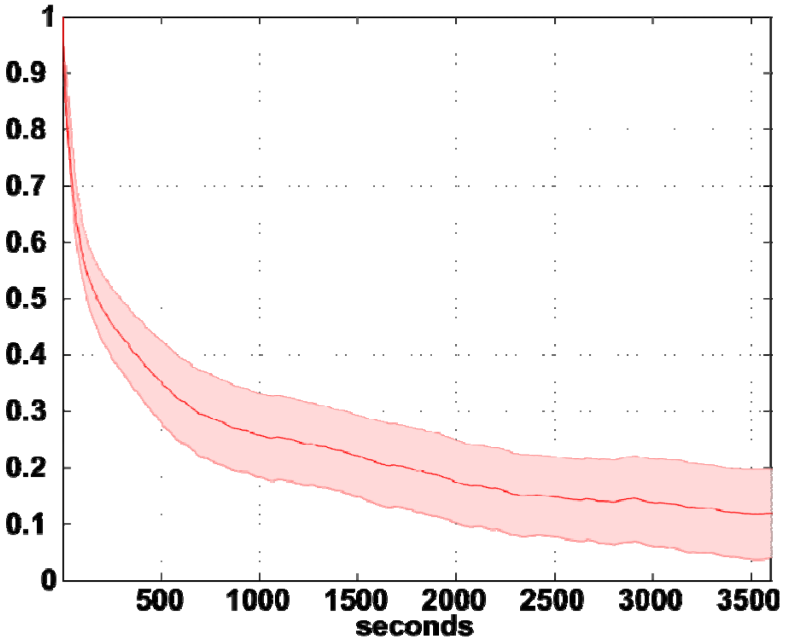
1
0.9
0.8
0.7
0.6
0.5
0.4
0.3
0.2
0.1
0
500
1000
1500
2000
2500
3000
3500
seconds

Figure S2

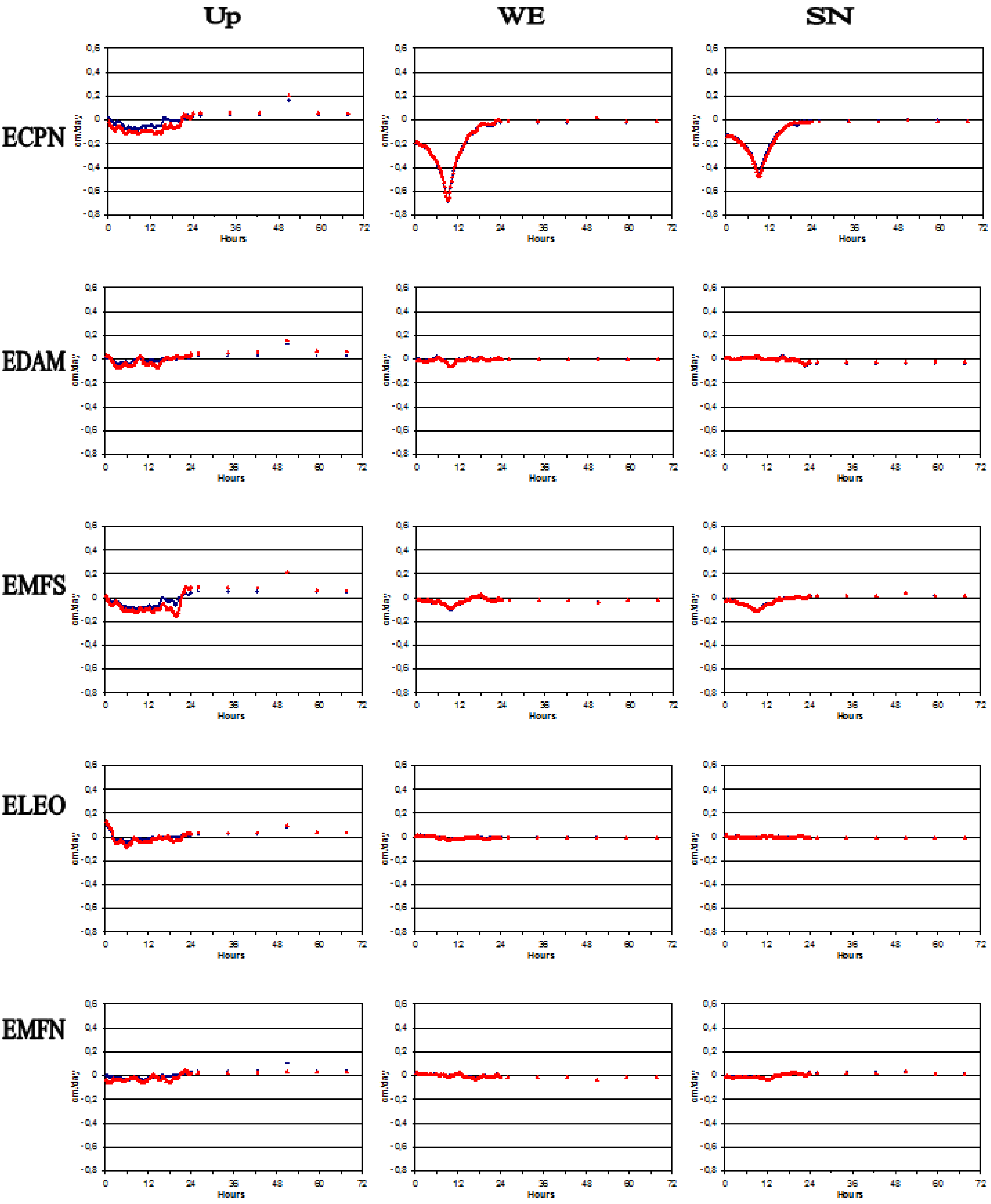
Up
WE
SN
ECPN
EDAM
EMFS
ELEO
EMFN
cm/day
Hours
0,6
0,4
0,2
0
-0,2
-0,4
-0,6
-0,8
0
12
24
36
48
60
72

Figure S3

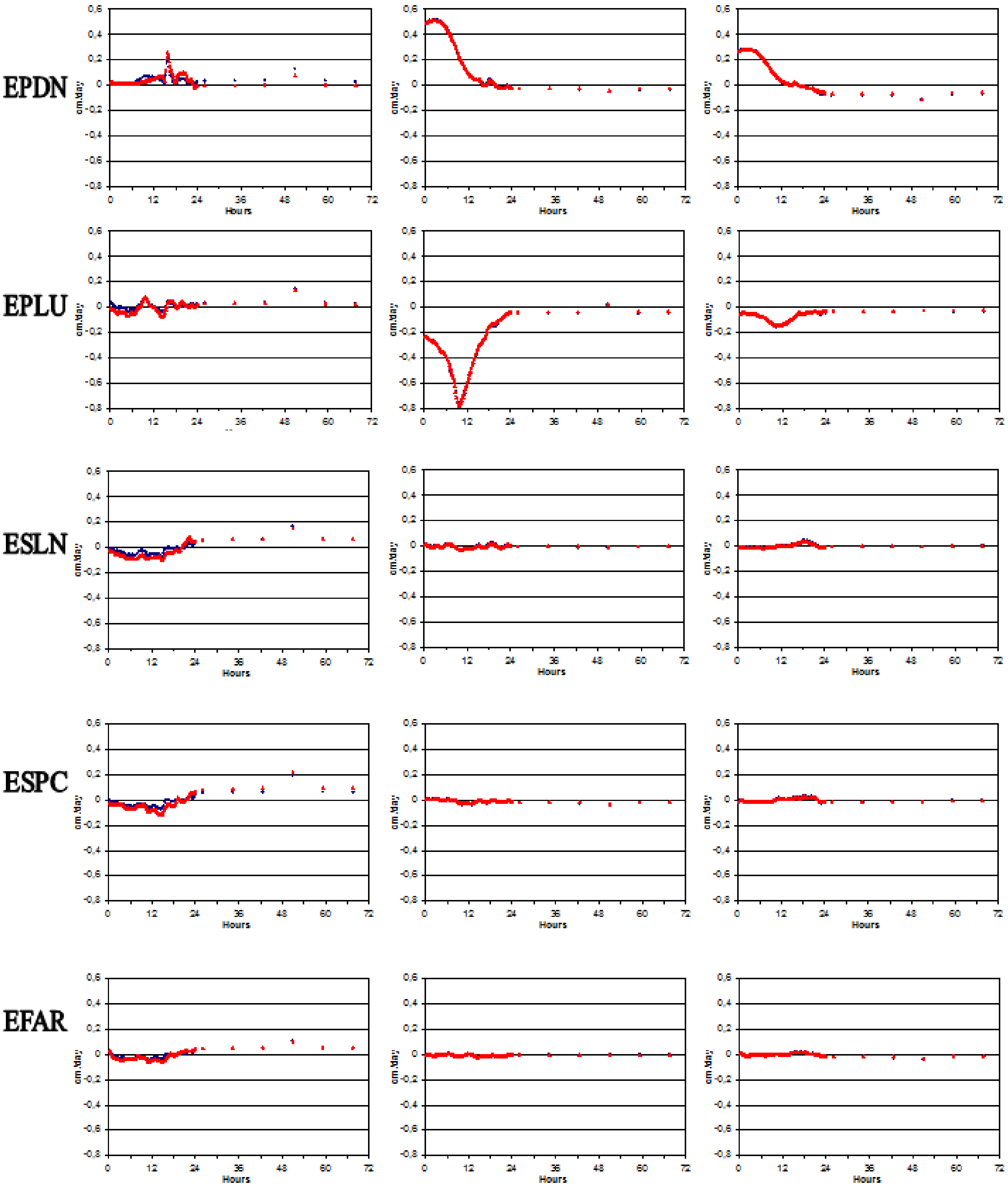

Figure S3 (continuation)

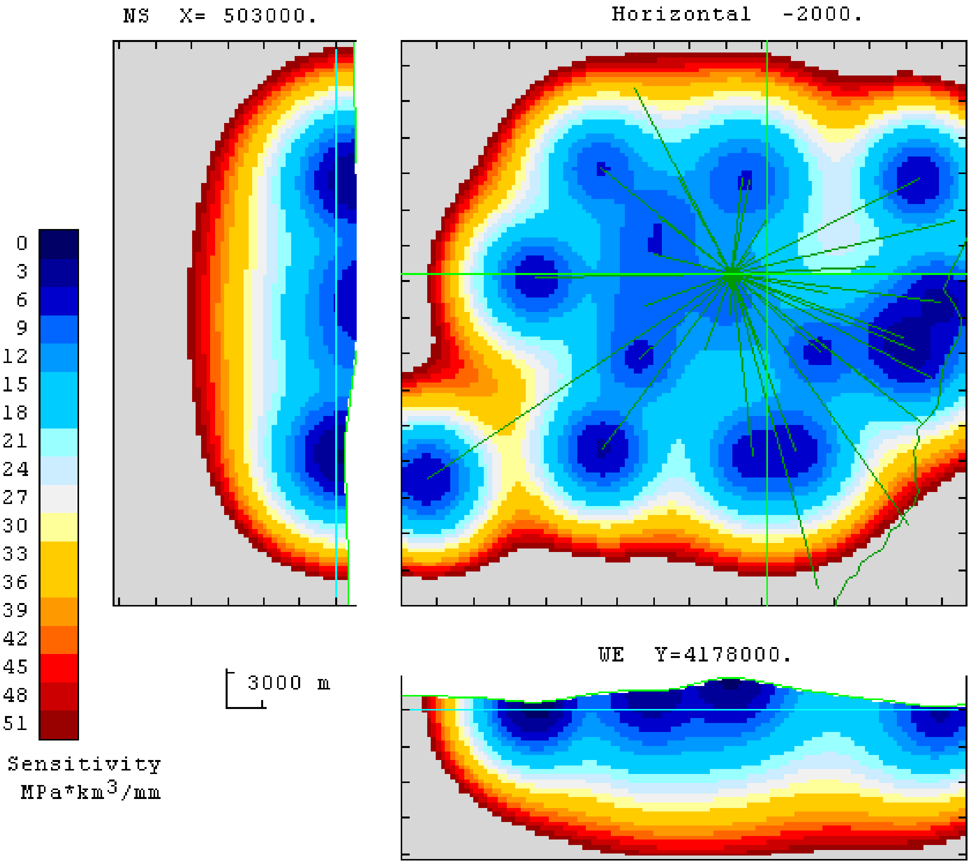
NS X= 503000.
Horizontal -2000.
WE Y=4178000.
3000 m
0
3
6
9
12
15
18
21
24
27
30
33
36
39
42
45
48
51
Sensitivity
MPa*km3/mm

Figure S4

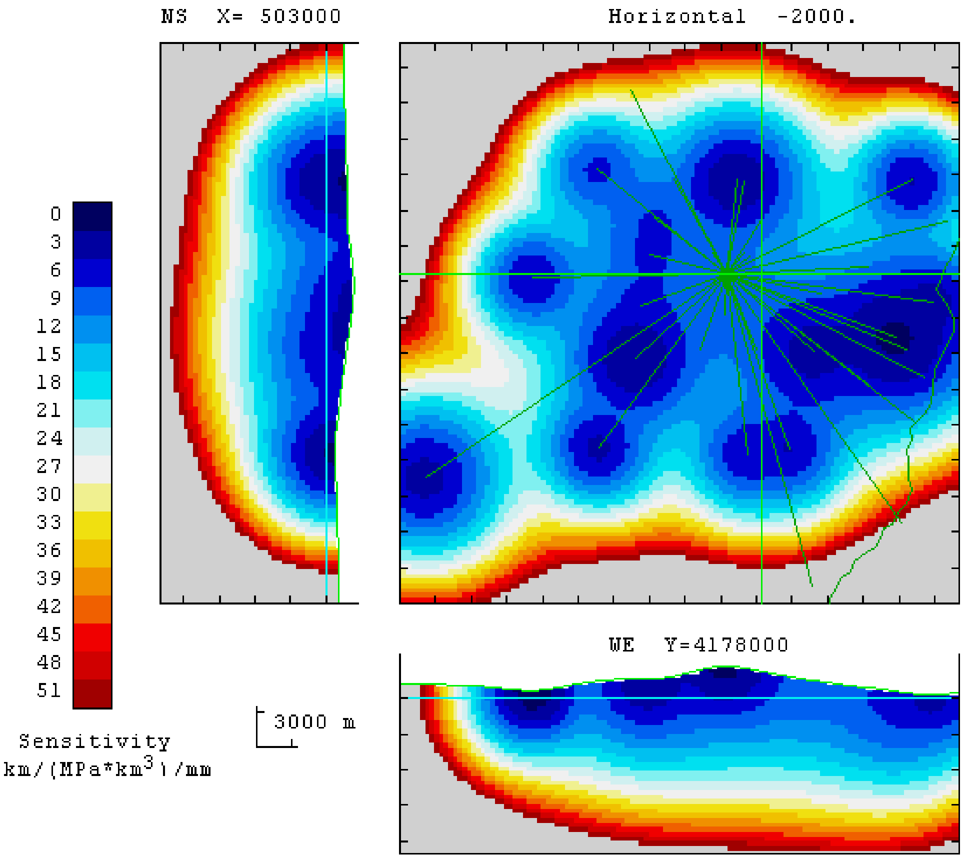

NS X= 503000
Horizontal -2000.
WE Y=4178000
0
3
6
9
12
15
18
21
24
27
30
33
36
39
42
45
48
51
Sensitivity
km/(MPa*km3)/mm
3000 m

Figure S5

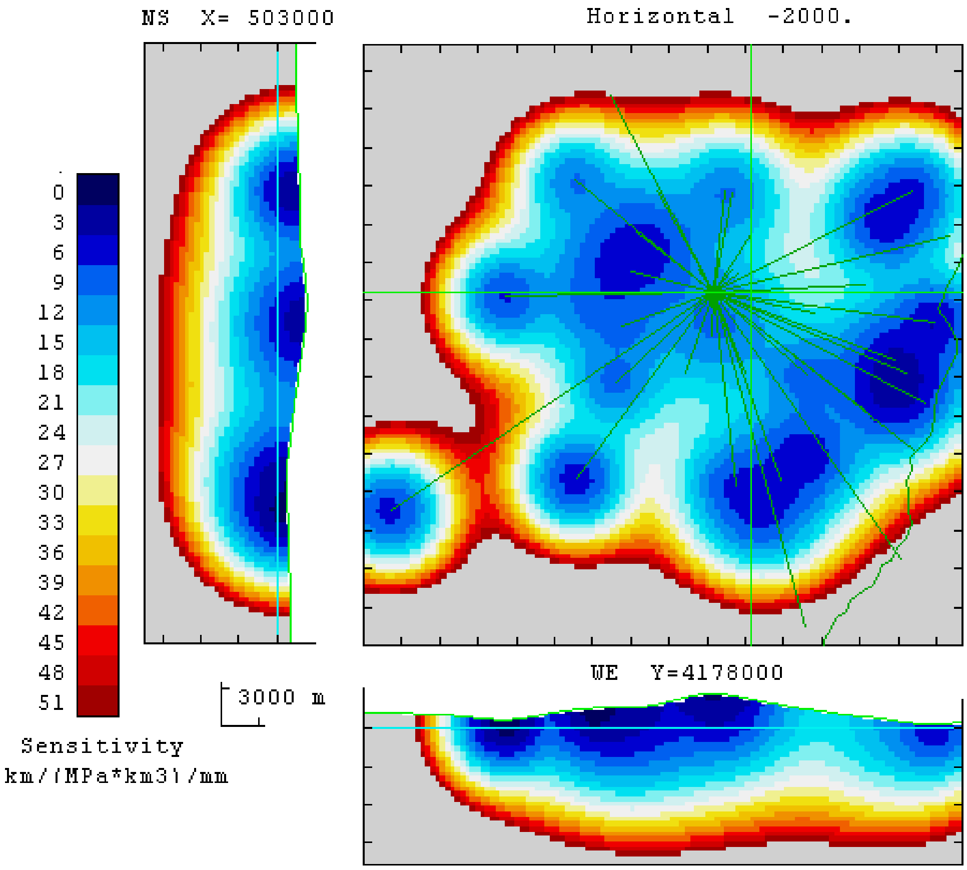
NS X= 503000
Horizontal -2000.
WE Y=4178000
3000 m
0
3
6
9
12
15
18
21
24
27
30
33
36
39
42
45
48
51
Sensitivity
km/(MPa*km3)/mm

Figure S6

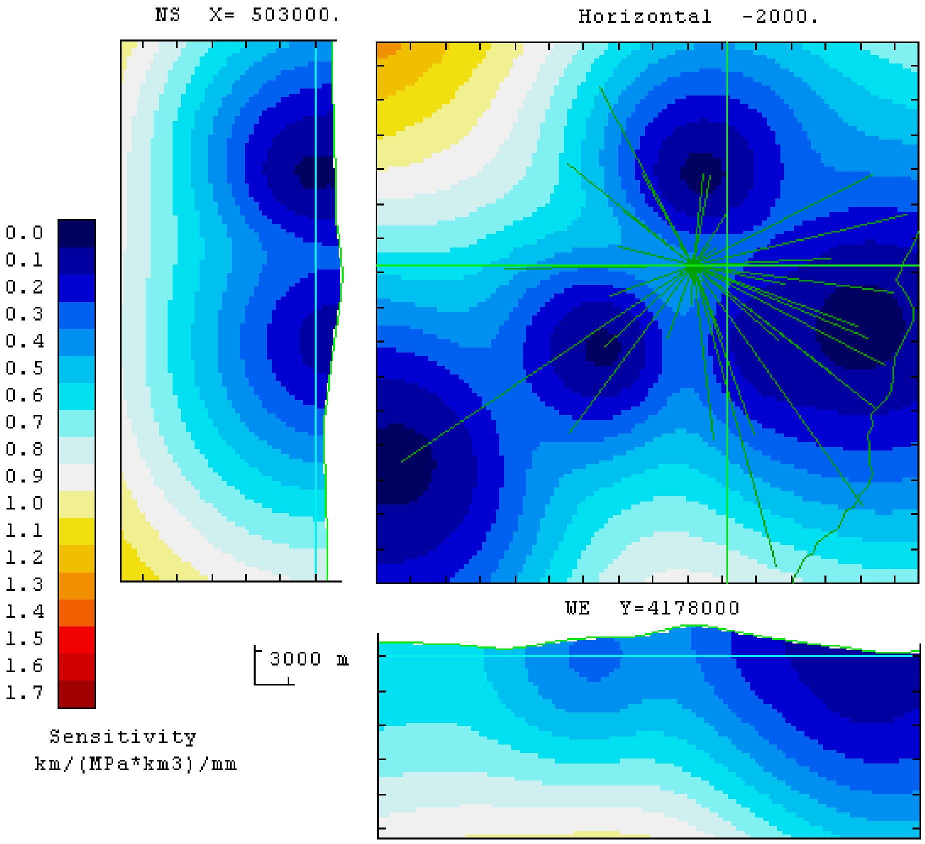
NS X= 503000.
Horizontal -2000.
WE Y=4178000
0.0
0.1
0.2
0.3
0.4
0.5
0.6
0.7
0.8
0.9
1.0
1.1
1.2
1.3
1.4
1.5
1.6
1.7
3000 m
Sensitivity
km/(MPa*km3)/mm

Figure S7

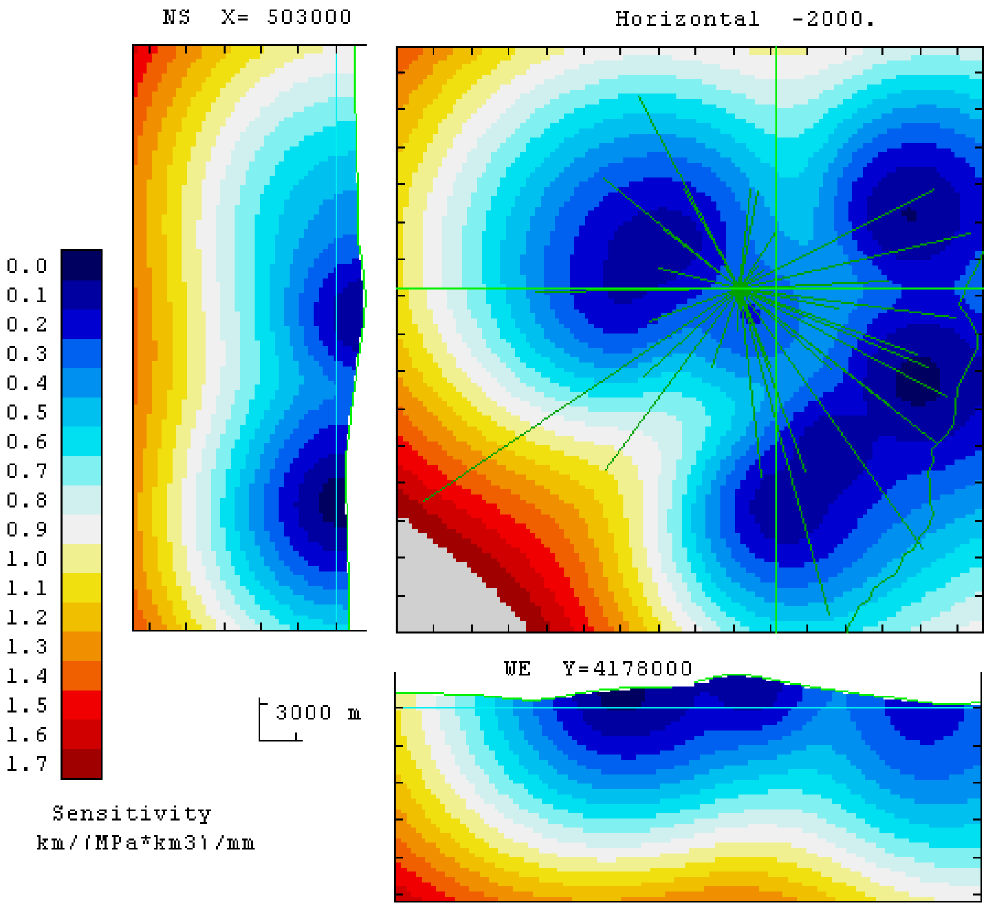
NS X= 503000
Horizontal -2000.
WE Y=4178000
3000 m
0.0
0.1
0.2
0.3
0.4
0.5
0.6
0.7
0.8
0.9
1.0
1.1
1.2
1.3
1.4
1.5
1.6
1.7
Sensitivity
km/(MPa*km3)/mm

Figure S8
